# Numerical simulations to predict complication after coarctation repair starting from 4D-Flow magnetic resonance imaging

**DOI:** 10.1007/s10554-026-03651-7

**Published:** 2026-03-30

**Authors:** Katia Capellini, Lamia Ait-Ali, Simone Garzia, Nicola Martini, Alberto Clemente, Angelo Monteleone, Massimiliano Cantinotti, Giuseppe Santoro, Pierluigi Festa, Simona Celi

**Affiliations:** 1 BioCardioLab, Bioengineering Unit, Fondazione G. Monasterio, Massa, Italy; 2 Institute of Clinical Physiology CNR, Massa, Italy; 3 Pediatric Cardiology and GUCH Unit, Fondazione G. Monasterio, Massa, Italy; 4 Diagnostic Imaging, Department of Radiology, Fondazione G. Monasterio, Massa, Italy

**Keywords:** Cardiac magnetic resonance, Aorta coarctation, 4D-Flow, Numerical simulations

## Abstract

Cardiac magnetic resonance (CMR) is recommended in post-aortic coarctation (Ao-Coa) repair. The combined use of computational fluid dynamics (CFD) simulations and CMR is a powerful instrument to estimate hemodynamic indices. We report a case of a complex Ao-Coa, operated by extra-anatomic conduit, with a pseudoaneurysm of the aortic arch. A 4D-Flow analysis and CFD simulation, performed starting from CMR acquired 6 years before, highlight an increase in flow complexity in the same site.

## Introduction

Aortic coarctation (Ao-Coa) is a congenital heart disease with a wide anatomical spectrum. Recent guidelines recommend cardiac magnetic resonance (CMR) follow-up in adult patients to monitor potential long-term complications as recurrent stenosis or aortic aneurysm [1]. Four-dimensional velocity mapping of flow (4D-Flow MRI) is an emerging tool for the evaluation of flow and velocities in any section of interest of the acquired volume. Through 4D-Flow MRI post processing, aorta wall shear stress (WSS) is qualitatively and quantitatively estimated [2] with a good reproducibility in intra and interobserver analysis [3]. However, it is well known that changes in spatial resolution affect WSS values and 4D-Flow MRI acquisitions with low spatial resolution, that are typically used in clinic, present higher WSS variability [4].

Recently, computational fluid dynamics (CFD) simulations based on 4D-Flow MRI allow the evaluation of hemodynamic parameters including WSS [5] with both high spatial and temporal resolution. Moreover, previous published studies speculated that abnormal hemodynamics and WSS might contribute to the risk of aortic dilatation [6]. Fluid dynamics of Ao-Coa was evaluated in different studies as well as the hemodynamic indicators after Ao-Coa repair to predict the efficacy of intervention and to investigate possible complications [7–9]. We report fluid dynamic characteristics in a complex Ao-Coa operated by an anatomical surgical bypass which developed an aortic pseudoaneurysm of the aortic arch at follow-up.

## Case report

**Fig. 1:**
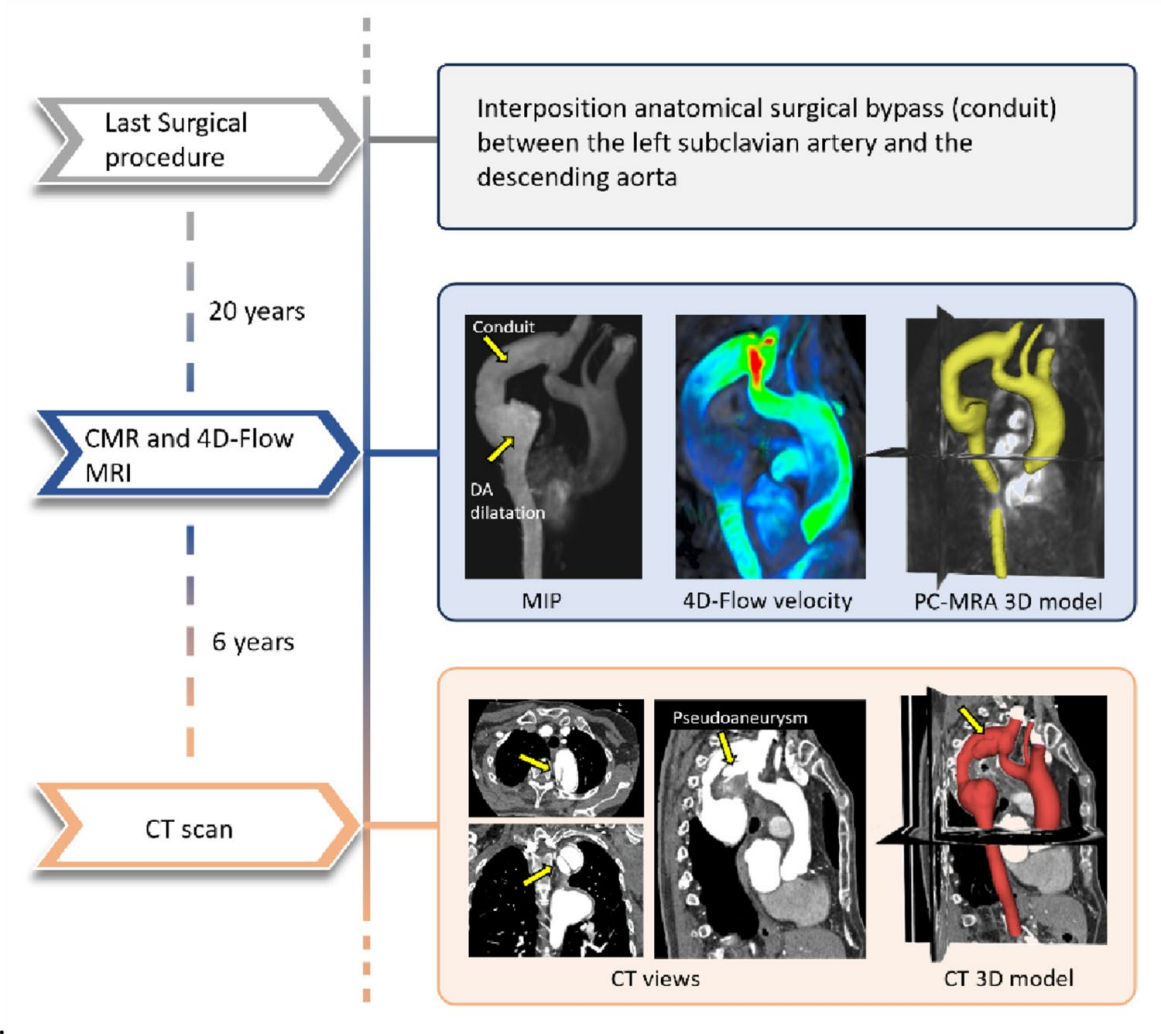
Case history timeline after complex aortic coarctation repair. The first arrow marks the last surgery. Twenty years later, CMR with 4D-Flow MRI shows: (i) MIP (Mean Intensity Projection), highlighting the patent conduit and distal anastomotic dilatation; (ii) 4D-Flow velocity map; (iii) PC-MRA overlaid on the 3D reconstructed model. Six years later, CT images in three standard views (i) demonstrate a pseudoaneurysm at the distal conduit–arch anastomosis, with the corresponding CT-based 3D reconstruction (ii)

A patient with complex Ao-Coa underwent several surgical procedures, the last one was an interposition anatomical surgical bypass between the left subclavian artery and the descending aorta. The chronological course of the patient’s surgical procedures and imaging follow-up described here is summarized in the timeline shown in Fig. 1. During follow-up, CMR examinations were performed; the one reported here was acquired 20 years after surgical repair and confirmed a patent conduit, a native aortic isthmus, and a dilatation at the distal conduit–descending aorta anastomosis. The CMR examination with gadolinium contrast was performed on a 3 Tesla scanner (Ingenia, Philips Medical Systems, the Netherlands) according to a standard clinical protocol. A 4D-Flow MRI sequence was also prescribed in sagittal orientation covering the entire aorta with the following parameters: velocity-encoding of 200 cm/s in three directions, reconstructed spatial resolution 1.95 × 1.95 × 2.5 mm, flip angle 15°, the repetition time (TR)/echo time (TE) 4.8/2.8 ms, 20 phases, temporal resolution of 39.6 ms, and a sensitivity encoding factor of 2 without respiratory gating.

Six years later, a CT scan was prescribed for the anatomical evaluation of the repaired coarctation and highlighted a new onset large pseudoaneurysm of the aortic arch at the level of the anastomosis with the conduit.

CT acquisition was performed with a 320-detector scanner (Toshiba Aquilon One, Toshiba, Japan), an iodinated contrast medium was adopted, and the dataset was characterized by a pixel size of 0.468 mm and a slice thickness of 1 mm.

The image datasets were retrospectively analysed to investigate the aorta hemodynamics during the Ao-Coa repair follow-up and assess the efficacy of CFD simulations to predict potential long-term complications starting from 4D-Flow MRI. The post processing of 4D-Flow MRI data was performed by a custom in-house software implemented in VMTK-Python, the 4D-Flow Phase Contrast Magnetic Resonance Angiography (PC-MRA) was computed and velocity patterns and WSS were evaluated [10]. Specifically, it was estimated from 4D-flow MRI by using a velocity-based linear extrapolation method, in which near-wall velocity gradients are estimated from adjacent voxels under the assumption of a linear velocity profile close to the wall, as previously described in [11].

3D models of the thoracic aorta before (3D-PRE) and after pseudoaneurysm formation (3D-POST) (Fig. 1) were reconstructed using 3D Slicer software. Both the 3D-PRE and 3D-POST models were segmented using a combination of threshold-based automatic segmentation and manual correction for complex regions, followed by surface smoothing with a low-pass Taubin filter to remove small artifacts. The 3D-PRE model was derived from the PC-MRA from the 4D-Flow MRI acquisition, while the 3D-POST model was reconstructed from follow-up CT images. As the vascular geometry and velocity field were both derived from the same 4D-Flow MRI dataset, they were inherently co-registered and shared the same spatial reference frame, allowing direct extraction of patient-specific inlet velocity profiles without additional rigid or non-rigid registration or interpolation procedures. The rigid registration between the 3D-PRE and 3D-POST models was performed solely for anatomical comparison and to identify, on the 3D-PRE geometry, the region corresponding to the site of subsequent pseudoaneurysm formation. CFD simulation was performed on the 3D-PRE model, to assess the potential role of hemodynamics in the later development of the pseudoaneurysm.

The computational domain was discretized using an unstructured volumetric mesh composed of tetrahedral elements in the core flow region and wedge elements in the near-wall boundary layers. A mesh sensitivity analysis was performed by systematically refining the mesh, and comparing the WSS and velocity results within the region corresponding to the site of subsequent pseudoaneurysm formation. Based on this sensitivity analysis, a mesh of approximately 1.52 × 10^6^ elements with an average edge size of 0.9 mm was selected, incorporating four near-wall layers with a growth factor of 1.2 and a total boundary layers thickness of 1.5 mm.

**Fig. 2:**
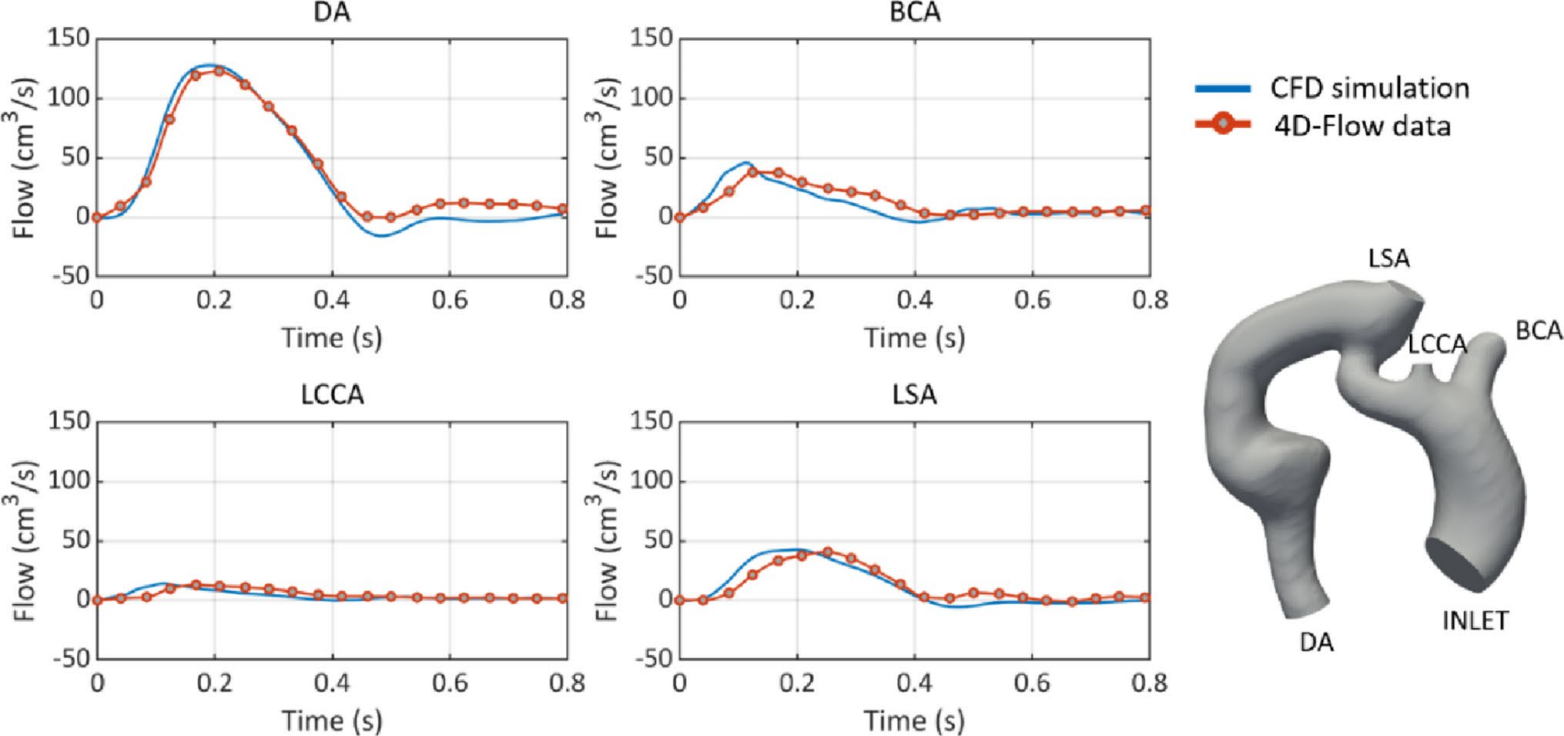
Comparison of flow profile extracted from 4D-Flow data and obtained from CFD simulation at the supra-aortic vessels and descending aorta

The wall was assumed rigid, and the blood was modelled as a Newtonian incompressible fluid, characterized by a density of 1060 kg/m^3^ and a constant viscosity of 0.0035 Pa·s. Blood flow was assumed to be laminar throughout the computational domain. Although the local Reynolds number reached values of up to 3680 at peak systole, these conditions were short-lived (0.1 s within a 0.8 s cardiac cycle), and Reynolds numbers were substantially lower during the remaining phases (below 2040). In pulsatile vessels, transition to turbulence requires not only high instantaneous Reynolds numbers but also sufficient temporal persistence and spatial development. Therefore, the adoption of a laminar flow model is considered consistent with the hemodynamic conditions investigated and with common practice in large-artery CFD under physiological conditions. The patient-specific 2D velocity profile extracted from the 4D-Flow MRI dataset at the aortic sinotubular junction was applied directly as the inlet boundary condition of the CFD model. At the outlets, patient-specific pressure boundary conditions were prescribed for the supra-aortic branches and the descending aorta using a three-elements lumped-parameter Windkessel model [12]. The Windkessel model was implemented through a user-defined function written in C language and compiled within ANSYS Fluent, allowing the boundary conditions to reproduce the patient-specific haemodynamic behaviour derived from clinical data. A comparison between CFD simulation results and 4D-Flow MRI analysis is reported in Fig. 2 in terms of flow profile in the same selected planes: at descending aorta (DA), at brachiocephalic artery (BCA), at left common carotid artery (LCCA) and at left subclavian artery (LSA). This evaluation revealed a qualitative good agreement between the patient’s flow profiles and the results obtained from CFD simulation.

**Fig. 3:**
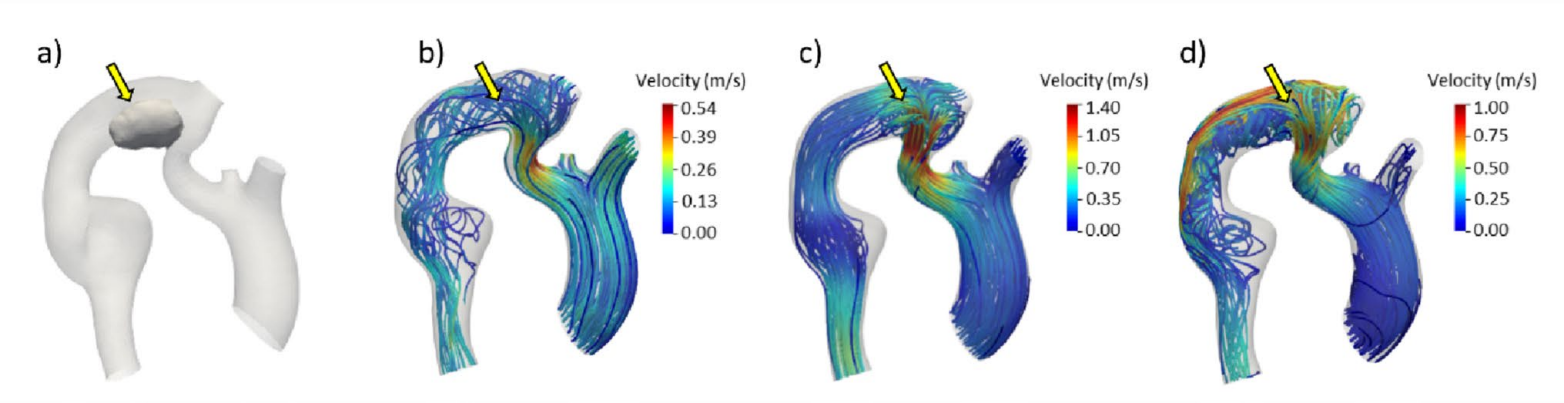
Focus on the region with the pseudoaneurysm (**a**), velocity streamlines at t1 (**b**), t2 (**c**) and t3 (**d**) instants of cardiac cycle

The blood flow behaviour was investigated through the evaluation of velocity streamlines in the aortic lumen, in the arch and pseudoaneurysm region for three different phases of cardiac cycle: maximum acceleration (t1), systolic peak (t2) and maximum deceleration (t3). Additionally, the WSS for each of selected phases of cardiac cycle was calculated to better highlight the potential effect of flow pattern on the arterial wall. The velocity patterns were characterized by an increase in flow complexity at the beginning of descending aortic region (Fig. 3) in correspondence with the tract where pseudoaneurysm was diagnosed six years after the CMR examination.

**Fig. 4:**
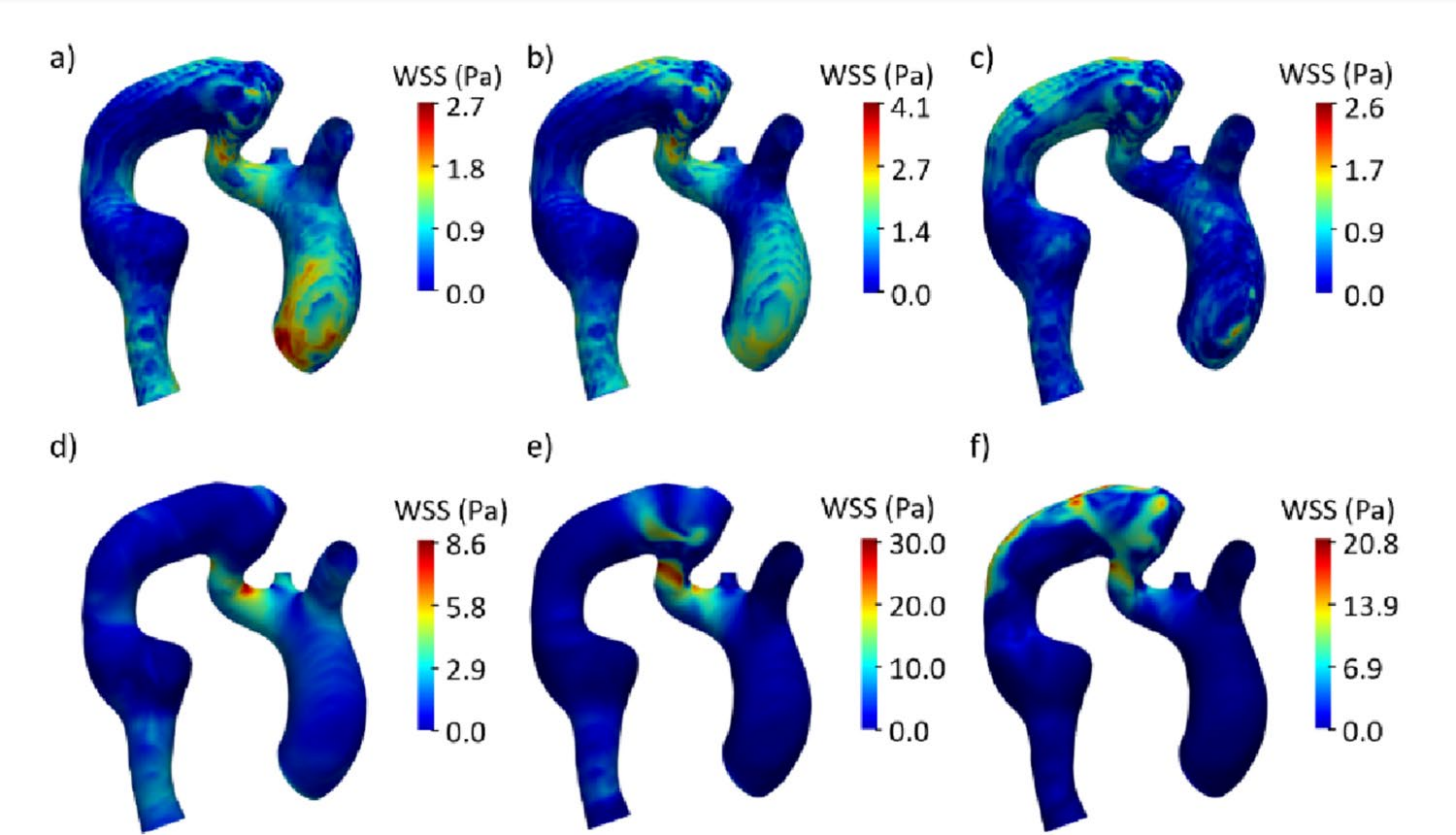
WSS distribution extracted from 4D-Flow data at t1 (**a**), t2 (**b**) and t3 (**c**) instants of cardiac cycle; WSS distribution obtained from CFD simulations at t1 (**d**), t2 (**e**) and t3 (**f**) instants of cardiac cycle

**Fig. 5:**
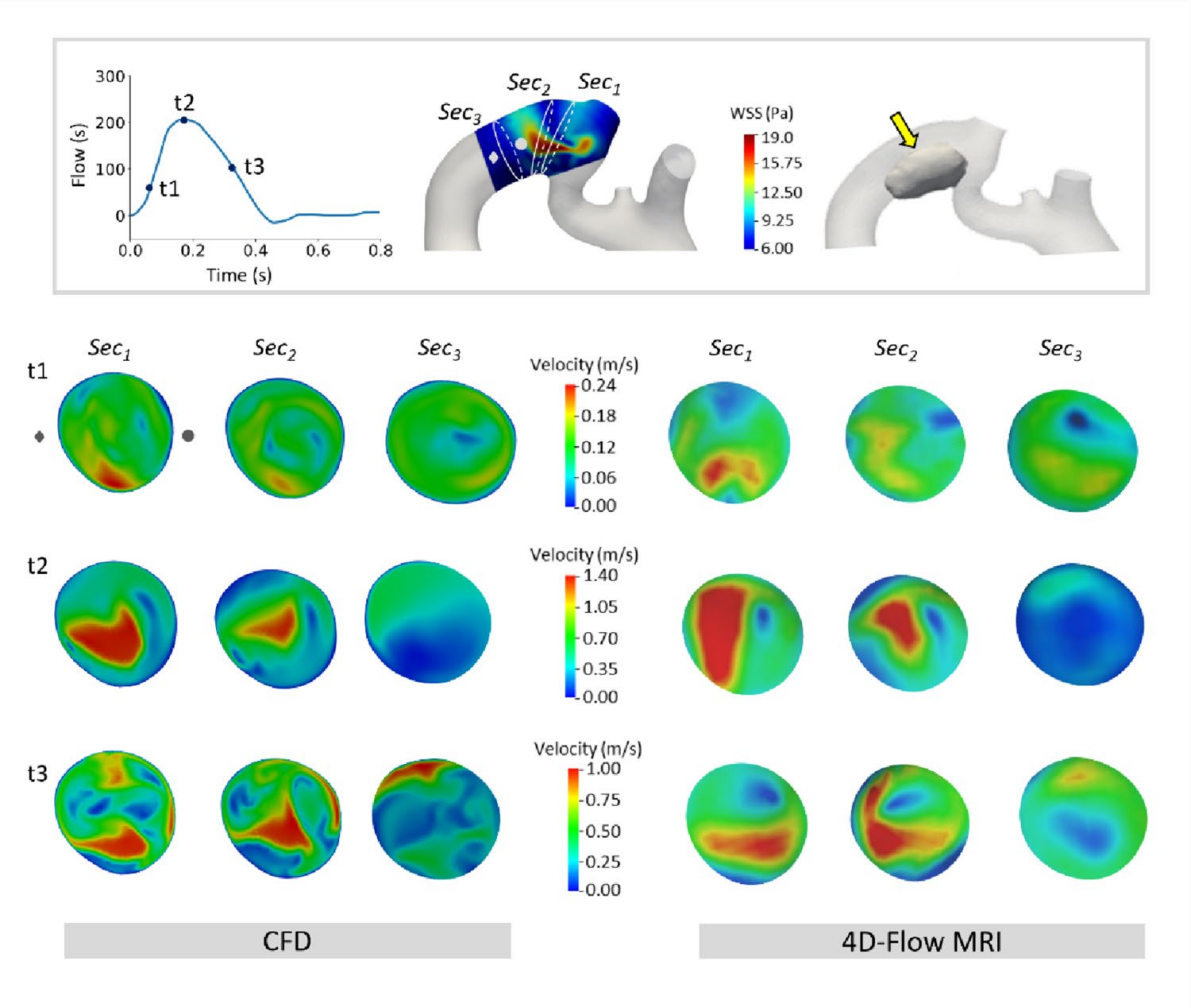
Focus on the CFD derived WSS values in the region corresponding to pseudoaneurysm formation at systolic peak; distributions of velocity at the three selected instants of cardiac cycle for three cross-sections in the region corresponding to pseudoaneurysm formation extracted from CFD and from 4D-Flow MRI data

The WSS distribution along the aorta extracted from 4D-Flow MRI and calculated by CFD simulations are reported in Fig. 4(a-c) and (d-f), respectively. In Fig. 5 the distributions of velocity derived from CFD and 4D-Flow MRI data are depicted for selected cross-sectional planes, along with a focus on the WSS values in the region corresponding to pseudoaneurysm formation.

## Discussion

Anatomical bypass repair is a surgical option in complex Ao-Coa [13] and aortic aneurysm is a potential complication of aortic coarctation surgery [14]. Accurate CFD simulations play a fundamental role in literature to extract hemodynamic indexes not measurable in-vivo; their power has increased significantly the more they are based on patient data in terms of geometry, boundary conditions as well as wall behaviour [15, 16].

We retrospectively analysed 4D-Flow MRI performed 6 years before the CT scan diagnosis of pseudoaneurysm. 4D-Flow MRI data coupled with CFD simulations were used to investigate velocity patterns and WSS in the thoracic aorta. A good qualitative agreement was observed between CFD-derived and 4D-Flow MRI velocity maps across the analysed aortic sections (Fig. 5). While absolute velocity values were comparable, minor differences in the spatial distribution of velocity were observed, especially during flow deceleration. These differences can be attributed to the limited spatial and temporal resolution of 4D-Flow MRI [18], whereas CFD simulations are able to capture localized flow features, instabilities, and enhanced secondary flow development, which become more pronounced during transient phases of the cardiac cycle. An underestimation of WSS by 4D-Flow MRI was observed (Fig. 4) due to its limited spatial resolution, in line with previously published studies [4, 17, 18]. The estimation of WSS from 4D-Flow MRI is inherently challenging because it relies on the accurate computation of near-wall velocity gradients, which are difficult to resolve with the available voxel size. Further sources of discrepancy arise from the use of a patient-specific geometry reconstructed from a temporally averaged PC-MRA dataset, while 4D-Flow MRI provides fully time-resolved velocity fields. For the MRI-based hemodynamic analysis, these time-resolved velocities are mapped onto a static vascular geometry, and this may introduce spatial mismatches between the measured velocities and the actual vessel wall location, particularly because the aorta undergoes motion and deformation throughout the cardiac cycle. Together, these factors can contribute to differences in both the spatial distribution and magnitude of WSS when comparing 4D-Flow MRI–derived estimates with CFD results. The hemodynamic analysis and numerical simulation revealed the presence of high velocities and high WSS at the site of the pseudoaneurysm with respect to physiological normal values reported in previous study on healthy subjects [19, 20]; moreover, an increase in flow complexity was also found in the same site as reported by the presence of small vortex structures near the artery wall (Fig. 3). Pseudoaneurysm development may be influenced by several factors such as local tissue fragility, suture-line degeneration, conduit material or geometry, infection, and blood pressure control and hemodynamics. With specific regard to hemodynamics, our results support the hypothesis that unfavourable WSS, and possibly complex flow patterns, may contribute to the risk of pseudoaneurysm formation at the anastomotic site and highlight the potential usefulness of 4D-Flow MRI in this population [21]. Further larger studies are warranted to confirm this hypothesis and to investigate usefulness of this approach to improve the surgical technique.

## Data Availability

The data that support the findings of this study are available from the corresponding authors upon reasonable request.
